# A field-measured dataset of plant and soil characteristics spanning six grassland types in the Zoige, northeastern Tibetan Plateau, China

**DOI:** 10.1038/s41597-026-07747-6

**Published:** 2026-06-26

**Authors:** Zhening Zhu, Yu Yin, Dexiong Xie, Wenliang Zhang, Wei Tang, Zizhi Wang, Wengui Wu, Shengxi Liao

**Affiliations:** 1 Institute of Highland Forest Science, Chinese Academy of Forestry, Kunming, 650233 China; 2 College of Forestry and Grassland, Nanjing Forestry University, Nanjing, 210037 China; 3 Sanjiang Group, Xining, 810003 China; 4 Yunnan Shangri-La Grassland Ecosystem Observation and Research Station, National Forestry and Grassland Administration, Chinese Academy of Forestry, Diqing, 674401 China

## Abstract

Grasslands, which cover nearly one-quarter of the Earth’s terrestrial surface, perform vital functions in carbon sequestration, biodiversity maintenance, and ecosystem stability. Zoige, situated on the northeastern Tibetan Plateau, hosts one of the world’s largest alpine peatland grassland complexes; however, quantitative field-based ecological datasets remain limited, constraining biomass evaluation, productivity estimation, and large-scale carbon assessment. To improve the availability of ecological data and enhance the accuracy of grassland resource characterization, this study provides a field-measured dataset from 80 sampling plots across six grassland types in Zoige between 2020 and 2022, which captures variations in community structure, soil nutrient status, dominant species composition, and biomass allocation patterns. The dataset includes geographic coordinates, sampling dates, species inventories, species records, vegetation richness and coverage, aboveground and belowground biomass, and soil attributes including moisture, bulk density, organic carbon, total nitrogen, and total phosphorus. The results indicate that the number of species in each grassland plot in Zoige ranged from 16 to 44. The mean (±SD) aboveground biomass, belowground biomass, and total biomass were 464.70 ± 286.52 g/m^2^, 2,517.57 ± 1,239.84 g/m^2^, 2,982.26 ± 1,409.94 g/m^2^, respectively. Significant correlations were observed among the biomass variables, with belowground biomass accounting for approximately 84.4% of the total biomass. The dominant species contributed substantially to community biomass, highlighting their important role in shaping ecosystem productivity. These relationships suggest that belowground biomass and dominant-species biomass may serve as useful indicators for estimating plot-scale biomass patterns in Zoige grasslands. This dataset provides a baseline reference for Zoige grassland ecosystem evaluation and supports future applications in carbon cycle accounting, biodiversity conservation, ecological modelling, and integration into global grassland data frameworks.

## Background & Summary

Grassland is among the largest terrestrial ecosystem, accounting for approximately one-quarter of the global land area^1,2^. Grassland ecosystems not only possess productive value by providing forage for animal husbandry and generating economic benefits but also play crucial ecological roles in terms of windbreak and sand fixation, water conservation, and soil and water retention^3,4^. Under the current goals of “carbon peaking” and “carbon neutrality”, grasslands play a pivotal role in ecological civilization construction. As significant carbon sinks, grasslands store 34% of the organic carbon in global terrestrial ecosystems^5,6^. Grassland ecosystems are expected to continue to function as carbon sinks for an extended period. However, grassland ecosystems are fragile and highly susceptible to external disturbances. Several factors, such as overgrazing, land-use change, and irrational land management can lead to land degradation, with statistics indicating that approximately 49% of global grasslands are experiencing varying degrees of degradation^7,8^. Therefore, comprehensively understanding grassland ecosystems and implementing targeted conservation measures are critically important.

The Tibetan Plateau is internationally recognized as a region with significant ecological value and high environmental sensitivity, and its high-elevation grassland ecosystems are highly vulnerable to climate change^9,10^. The Zoige area, located on the northeastern edge of the Tibetan Plateau, is the largest peat bog area in China and the largest alpine bog area in the world^11^. The area is also crucial for water conservation and water recharge in the upper reaches of the Yellow River. It is classified as a restricted development zone, is a key area for global biodiversity and is sensitive to global climate change^12^. Therefore, conducting systematic research in this region holds significant scientific value. Although numerous ecological studies have been conducted in the Zoige region in recent years, many of them have focused primarily on wetland ecosystems^13,14^, while relatively few studies have comprehensively addressed grassland ecosystems. Existing grassland-related studies have concentrated mainly on specific topics, such as grassland degradation, grazing impacts^15,16^, or particular ecological components^17^. Consequently, regionally integrated and multi-dimensional field datasets for grassland ecosystems remain relatively limited. This limitation constrains the ability of currently available data to comprehensively characterize regional ecological patterns and reduces their applicability for ecological modelling and large-scale ecosystem analyses.

In this study, we report a comprehensive grassland ecosystem dataset derived from systematic field investigations across 80 representative plots in Zoige. The sampling design strategically incorporated the spatial distribution patterns of grassland types and altitudinal gradients. The dataset systematically documents six grassland types (marsh meadow, wetland meadow, subalpine meadow, subalpine shrub meadow, alpine meadow, and marsh wetland), with each plot containing three core data modules: (1) habitat characteristics of the sample site (altitude, slope, aspect, mean annual temperature and mean annual precipitation); (2) grassland biomass (above ground biomass and below ground biomass), including the biomass of the top four dominant species; (3) soil physical and chemical properties (water content, bulk density, organic carbon, total nitrogen, total phosphorus, etc.). In addition, in this dataset, we used a standardized quality control scheme, which provides not only a baseline reference for regional ecological research but also data support for the global terrestrial ecosystem database.

## Methods

### Research site selection and basic site information

**Fig. 1:**
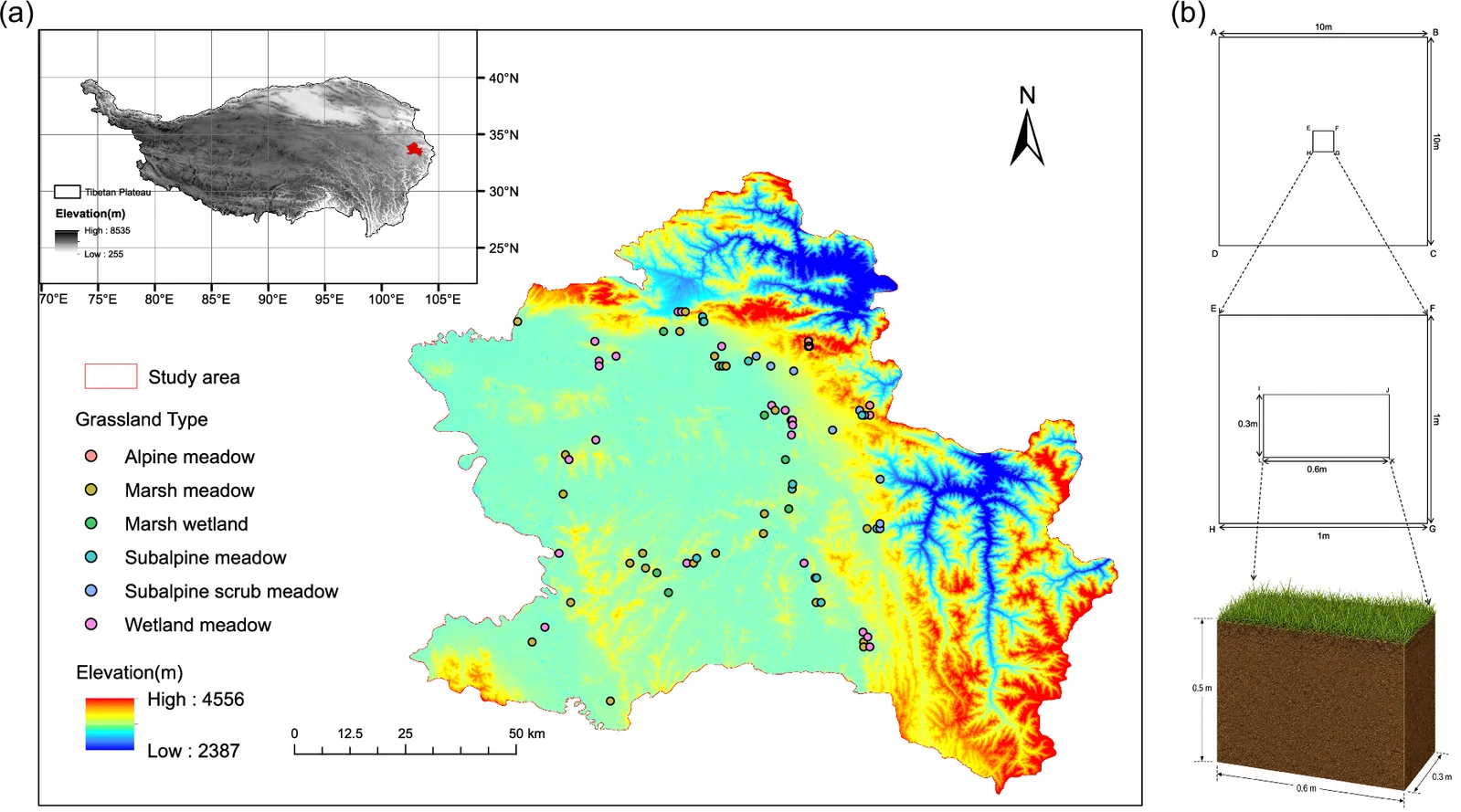
(**a**) Map of the study area and sampling sites (total number of site = 80). (**b**) Diagram of the sampling design for this study.

The study area is located on the northeastern Tibetan Plateau(Fig. 1a), with an area of 10,620 km^2^ and an average elevation of 3,500 m. Based on the Sichuan Province Grassland Resource Survey Map and the Zoige Country Grassland Type Map, and combined with the grassland classification used in recent Zoige studies^18^, six grassland types were finally identified: alpine meadow, marsh meadow, marsh wetland, subalpine meadow, subalpine scrub meadow, and wetland meadow. The selected sample plots represent grasslands at different elevations and topographic conditions. When setting up sample plots, the minimum spacing between plots should be determined comprehensively based on the basis of topography and plant communities heterogeneity, if the topography is similar and there are significant differences in plant communities, the spacing between plots should not be less than 200 m; if the plant communities differences are not significant, the spacing between plots should be greater than 2,000 m. When the topography is undulating, the sample plots should be surveyed every 100 meters of elevation difference. Considering survey routes, accessibility, and environmental heterogeneity, a total of 80 plots were established along regional elevation and vegetation gradients^19,20^. The topography in Zoige is complex and diverse. As shown in Fig. 1a, the landform exhibits a clear transition zone, where the central and western parts have broad and relatively flat plateaus, whereas the eastern and northern parts have rugged and steep mountains. Unstable surfaces and inconvenient transportation make it difficult to establish standardized sample plots. Furthermore, the vegetation cover in this area is low, and there are no complete and representative grassland types, resulting in a sparse distribution of sample plots. The Zoige Marsh is the world’s largest high-elevation marsh area. During the peak grassland growing period, parts of the Zoige region become seasonally waterlogged because of abundant rainfall and swamp development, increasing the difficulty of field surveys. Therefore, the sampling sites were preferentially selected in areas with stable surface conditions while maintaining the representativeness of regional grassland types. The study area has a plateau cold temperate humid monsoon climate, with an average annual temperature of 1.1°C and an annual precipitation of 648.5 mm. The soil types are diverse and mainly consist of marsh soil and subalpine meadow soil^21^. The unique geographical and climatic conditions of this region have created a diverse range of grassland resources and types, with marsh meadow and wetland meadow being the main grassland types.

### Experimental design

Field surveys of the sample plots were conducted in August every year from 2020 to 2022, when the grassland is at its peak biomass^22,23^. In addition, a supplementary survey was carried out in August 2024 to revise the species classification. We established 10 m × 10 m plots (Fig. 1b ABCD) to investigate the species composition of grassland in the area and selected 1 m × 1 m quadrats (Fig. 1b EFGH) within each plot for sampling. The plots were randomly distributed at different elevations (3,200–3,900 m), while survey accessibility was considered. Adjacent plots were established either in different grassland types or at different elevation levels. Furthermore, to ensure ecological representativeness, our sampling strategy prioritized relatively intact habitats with limited visible human disturbance, stable species compositions, and consistent biomass levels, covering most regional landform types (excluding aquatic environments). The sampling sites were selected away from major roads, settlements, and other areas with intensive anthropogenic activities whenever possible.

During the survey, we used portable GPS devices (Magellan eXplorist 510) to record the latitude, longitude, and altitude of each sampling point, together with basic topographic information such as slope and aspect. In addition, we recorded detailed vegetation attributes within each quadrat, including the average plant height, abundance, dominant species, species richness, and canopy area. Destructive sampling methods were subsequently employed to collect plant biomass within the quadrats. According to previous studies^24^, the most suitable sampling size is a sample within an area of 0.5 m × 0.5 m. Compared with other sampling areas, such as the 1 m² square (1 m × 1 m), 0.5 m² rectangle (0.5 m × 1 m), 0.25 m² circle (0.28 m in diameter) and 0.09 m² square (0.3 m × 0.3 m), the cumulative coefficient of variation of this area is smaller^25^. However, because large areas of the Zoige are dominated by marsh meadow and wet meadow with water-saturated and unstable soils, plant roots are tightly interwoven and difficult to separate completely during biomass collection. The cohesive peat layer further increases the effort required for harvesting and processing, while the high water table complicates sample handling in the field. For practical, reproducible, and safe sampling, we delineated a 0.3 m × 0.6 m harvest area within each plot (Fig. 1b IJKL) and converted the measurement results to estimate the biomass for a standard 1 m × 1 m quadrat. Within each harvest area, we excavated the soil to a depth of approximately 50 cm, which contains more than 95% of the root biomass in the alpine meadow ecosystem. The excavated soil clods were carefully washed in a pool or along a stream to separate plant roots from soil particles and organic debris. Dead roots and non-plant material were removed as much as possible, and only the live root biomass was retained for analysis. After sampling, all the plants in the quadrat were classified, photographed, and identified by plant experts. When the aboveground and underground biomass of plants is separated, the boundary between green or light-green tissues and non-green tissues of plant’s root and stem was defined as the dividing line. All the plant organs (such as stems, leaves and petioles) above this boundary were classified as the aboveground biomass, whereas the root tissues without green pigment below the boundary were categorized as the underground biomass. The plant samples were cut along the dividing line using scisssors to separate the aboveground and underground parts. After air drying, the wet masses of the aboveground and belowground parts of the various plants were determined using a high-precision electronic balance^23^. The weighed samples were placed in envelopes, labelled, and taken back to the laboratory for verification to confirm the accuracy of the names. After being returned to the laboratory, the fresh samples were dried in an oven at 65°C until they reached a constant weight, after which the dry mass of the aboveground and belowground parts of each sample was recorded^26^, and the biomass data of the samples were recorded.

After the surface litter was removed, soil samples were collected from each plot using a 100 cm^3^ ring sampler (5 cm depth) at two depth intervals (0–10 cm and 10–20 cm). These samples were stored in labelled containers and then brought back to the laboratory for analysis.Two of the ring samples from different soil layers were oven-dried to determine the soil bulk density and soil moisture content. The average value was used to determine the bulk density and moisture content of the soil from the other two ring samples. The soil from the other two ring samples from different soil layers was mixed, the plant roots and gravel were removed, and the samples were air-dried, and then passed through a 2 mm sieve (80 mesh). The processed samples were used to determine soil organic carbon (SOC), total nitrogen (TN), total phosphorus (TP), and pH. SOC was measured using the K_2_Cr_2_O_7_-H_2_SO_4_ external heating oxidation method, TN was determined by the Kjeldahl digestion method, and TP was quantified using the molybdenum antimony colorimetric method^27^. Soil pH was measured with a calibrated pH meter after extraction with distilled water for 30 minutes at a soil-to-water ratio of 1:2.5^28^. Climate variables were derived from the ERA5-Land reanalysis dataset generated by the Copernicus Climate Change Service, which is available from the Copernicus Climate Data Store (https://cds.climate.copernicus.eu/datasets/reanalysis-era5-land?tab = overview)^29^. We downloaded and processed the annual climate data corresponding to the study period to calculate various climate variables. The climate-related data extracted from each plot corresponds to the annual average of the sampling year, as the grassland growth cycle is not confined to a single point in time but is a long and complex process, thus better representing the climatic conditions at the time of sampling.

## Data Records

The dataset is available at Figshare (https://doi.org/10.6084/m9.figshare.32556594)^30^ and (https://doi.org/10.6084/m9.figshare.32553978)^31^. This section describes the content of the dataset in detail. The dataset contains two files:

### Dataset (i): Zoige grassland_study sites

This file describes comprehensive field observation data for each sampling site (Table 1). The sampling site information included site ID, plot area, grassland type, sampling date, latitude and longitude; the landform information includes elevation, aspect, and slope; the climatic information included mean annual temperature, mean annual precipitation, and solar radiation; the soil information included soil organic carbon, soil water content, soil bulk density, total nitrogen, total phosphorus, and soil pH; the biomass information included aboveground biomass, belowground biomass, total biomass, and dominant species biomass for each sampling site; and the vegetation information included coverage, root–shoot ratio, species richness, and complete species list within the plot^30^.

**Table 1:** Descriptive statistics of the factors used in this database.

Category	Variable name	Abbreviation	Variable type	Measurement method	Unit/format
Information	Site ID		factor	defined	
	Plot area		factor	defined	1 m × 1 m
	Grassland type		factor	defined	
	Sampling date		factor	defined	YY/MM/DD
	Latitude	LAT	numeric	logged	°
	Longitude	LON	numeric	logged	°
Landform	Elevation	ELE	numeric	logged	m
	Slope	Slope	numeric	logged	m/100 m
	Aspect	Aspect	numeric	logged	°
Climate	Mean annual temperature	MAT	numeric	logged	°C
	Mean annual precipitation	MAP	numeric	logged	mm
	Solar radiation	SOL	numeric	logged	MJ/m^2^/year
Soil	Soil organic carbon	SOC	numeric	logged	g/kg
	Soil water content	SWC	numeric	logged	%
	Soil bulk density	SBD	numeric	logged	g/cm^3^
	Soil total nitrogen	TN	numeric	logged	g/kg
	Soil total phosphorus	TP	numeric	logged	g/kg
	Soil pH	pH	numeric	logged	
Biomass	Aboveground biomass	AGB	numeric	logged	g/m^2^
	Belowground biomass	BGB	numeric	logged	g/m^2^
	Total biomass	TB	numeric	logged	g/m^2^
	Dominant species biomass	DSB	numeric	logged	g/m^2^
Vegetation	Coverage	Cover	numeric	logged	%
	Root-Shoot ratio	R/S	numeric	logged	
	Species richness	SR	numeric	logged	
	Species list	SP_list	text	logged	

### Dataset (ii): Zoige grassland_dominant species biomass analysis results

This file provides relevant information on the dominant species at each sample site(Table 2), including the aboveground biomass, belowground biomass, total biomass and root-to-crown ratio information of the dominant species (the top four species in terms of biomass). Additionally, it includes the proportions of the cumulative biomass of the top 1–4 dominant species relative to the total biomass of each sampling site^31^.

**Table 2:** Descriptive statistics of the factors used in this database.

Category	Variable name	Abbreviation	Variable type	Measurement method	Unit/format
Information	Site ID		factor	defined	
	Grassland type		factor	defined	
Biomass	Aboveground biomass	AGB	numeric	logged	g/m^2^
	Belowground biomass	BGB	numeric	logged	g/m^2^
	Total biomass	TB	numeric	logged	g/m^2^
Dominant species biomass	Dominant species Latin name	DSP_name	factor	defined	
	Dominant species aboveground biomass	DSP_AGB	numeric	logged	g/m^2^
	Dominant species belowground biomass	DSP_BGB	numeric	logged	g/m^2^
	Dominant species total biomass	DSP_TB	numeric	logged	g/m^2^
	Dominant species Root-Shoot ratio	DSP_R/S	numeric	logged	
Dominant species biomass ratio	Dominant species aboveground biomass ratio	DSP_AGB ratio	numeric	logged	
	Dominant species belowground biomass ratio	DSP_BGB ratio	numeric	logged	
	Dominant species total biomass ratio	DSP_TB ratio	numeric	logged	

### Grassland type

**Fig. 2:**
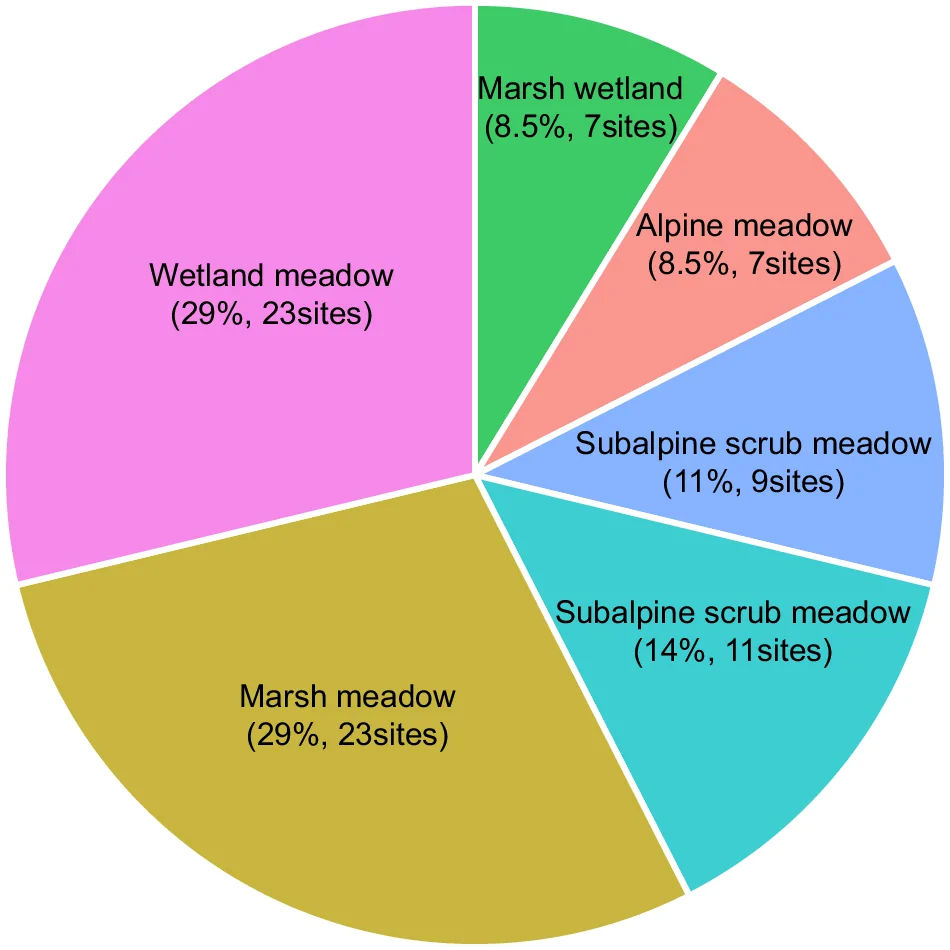
Composition of grassland types in the Zoige.

The dataset includes 80 plots in Zoige, covering six grassland types: subalpine meadow, marsh meadow, subalpine scrub meadow, wetland meadow, alpine meadow, and marsh wetland (Fig. 2); the classification criteria for grassland types are shown in Table 3. A total of 2,314 plant occurrences were recorded, representing 428 species belonging to 39 families and 160 genera. Overall, the biomass indicators varied significantly across the different grassland types. The aboveground biomass ranged from 76 g/m^2^ to 1285 g/m^2^, whereas the belowground biomass was significantly greater, ranging from 531 g/m^2^ to 5,978 g/m^2^. Therefore, the total biomass fluctuates within a wide range of 700 g/m^2^ to 6,740 g/m^2^, with wetland meadow and marsh meadow generally exhibiting relatively high biomass levels. The dominant species biomass tended to be consistent with the total biomass, which accounted for a significant proportion of the total biomass in some of the marsh meadow and wetland meadow plots. The root–shoot ratio is generally high, ranging from 1 to 12 in most plots and reaching as high as 18 in some areas, indicating a strong tendency for belowground carbon allocation in the grassland vegetation of the study area. The species richness varied between 16 and 44 species, while the vegetation cover was generally high, exceeding 0.80 in most plots, reflecting the relatively dense grassland structure and high productivity in the Zoige.

**Table 3:** Main characteristics of the six grassland types in the Zoige.

Grassland type	Definition	Key environmental characteristics	Dominant species and main associated plants
Marsh wetland	Mainly distributed at the inner margin of low-moisture meadows, the outer edge of marshes, valley bottoms, terraces, and seasonally or permanently flooded depressions	Extremely wet; soils are permanently or seasonally waterlogged with a high groundwater table	*Blysmus sinocompressus*, *Carex setschwanensis*, *Carex tibetikobresia*, *Carex muliensis*, *Carex alatauensis*, *Carex enervis*, and *Carex littledalei*
Marsh meadow	Occurs in low-lying areas of rivers and lakes, broad valley bottoms, and groundwater discharge zones at the front of alluvial fans; characterized by thick humus and a high groundwater table	Very moist, with seasonal or locally permanent waterlogging; dominated by peat meadow soils and meadow marsh soils	*Blysmus sinocompressus*, *Carex setschwanensis* and *Elymus nutans*
Wetland meadow	Widely distributed and mosaically interspersed with other grassland types; generally wetter than typical meadows but not waterlogged	Moist but not inundated; represents a transition zone between marshy vegetation and upland meadows	*Carex enervis*, *Blysmus sinocompressus*, *Carex setschwanensis*, *Carex littledalei*, *Carex capillifolia* and *Elymus nutans*
Subalpine meadow	Occurs in the transition zone between forest and grassland, typically on subalpine hills, mountain slopes, and flatlands; vegetation cover > 80%	Moderately moist, located in the subalpine climatic zone	*Carex coninux*, *Carex crebra*, *Carex setschwanensis*, *Blysmus sinocompressus*, *Elymus burchan-buddae*, *Brachypodium sylvaticum* and *Carex capillifolia*
Subalpine scrub meadow	Distributed on mountain and subalpine sunny slopes and along riverbanks; characterized by the coexistence of shrubs and herbs	Moderately dry; sunny slopes provide relatively warm and dry microclimates; affected by shrub invasion	*Blysmus sinocompressus*, *Carex setschwanensis*, *Carex kokanica*, *Carex parvula*, *Carex coninux* and *Carex capillifolia*
Alpine meadow	Occurs above 3,700 m on mountain tops, slopes, and valleys in high-cold environments	Low temperatures, strong winds; soils are alpine meadow soils rich in organic matter and moderately acidic to slightly alkaline	*Carex parvula*, *Carex capillifolia*, *Carex coninux* and *Bistorta vivipara*

In reference to previous literature, species whose relative abundance is greater than 5% are defined as dominant species, those whose relative abundance is between 1% and 5% are defined as common species, and those whose relative abundance is less than 1% are defined as rare species. Dominant species are characterized by relatively high abundance or biomass within plant communities and are often associated with important contributions to community structure and biomass composition^32^. In this study, six grassland types were identified in the Zoige region, each of which exhibited distinct dominant species compositions. (1) The marsh meadow was dominated by *Blysmus sinocompressus*, which was accompanied by *Carex setschwanensis* and *Elymus nutans*. (2) *Carex enervis*, *Blysmus sinocompressus* and *Carex setschwanensis* played a leading role in wetland meadow, with *Carex littledalei*, *Carex capillifolia* and *Elymus nutans* also playing important roles. (3) In the subalpine meadow, *Carex coninux* was the leading contributor, followed by *Carex crebra*, *Carex setschwanensis*, *Blysmus sinocompressus*, *Elymus burchan-buddae*, *Brachypodium sylvaticum* and *Carex capillifolia*. (4) In subalpine scrub meadow, *Blysmus sinocompressus*, *Carex setschwanensis*, *Carex kokanica*, *Carex parvula*, *Carex coninux* and *Carex capillifolia* played important roles. (5) In the alpine meadow, *Carex parvula* and *Carex capillifolia* were identified as the principal dominant species, accompanied by *Carex coninux* and *Bistorta vivipara*. (6) In the marsh wetland, *Blysmus sinocompressus* was the dominant contributor, with *Carex setschwanensis*, *Carex tibetikobresia*, *Carex muliensis*, *Carex alatauensis*, *Carex enervis* and *Carex littledalei* also playing important roles. Across different grassland types, species of Carex and Blysmus consistently dominated the plant communities, with *Carex setschwanensis* and *Blysmus sinocompressus* emerging as the most widespread and influential species across multiple grassland types.

### Dominant species

In this study, a total of 16 dominant plant species were recorded in the grassland communities, with distinct differences in biomass contributions among the species. The ranking of the dominant species revealed that Cyperaceae and Poaceae plants were predominant across all the grassland types in Zoige, with Cyperaceae still accounting for the majority of the biomass, which is represented mainly by the genera *Carex* and *Blysmus*. Cyperaceae constitute the structural and functional basis of grasslands, which exhibit strong adaptability to the alpine environment of Zoige and maintain both grassland productivity and soil stability even under cold conditions.

**Fig. 3:**
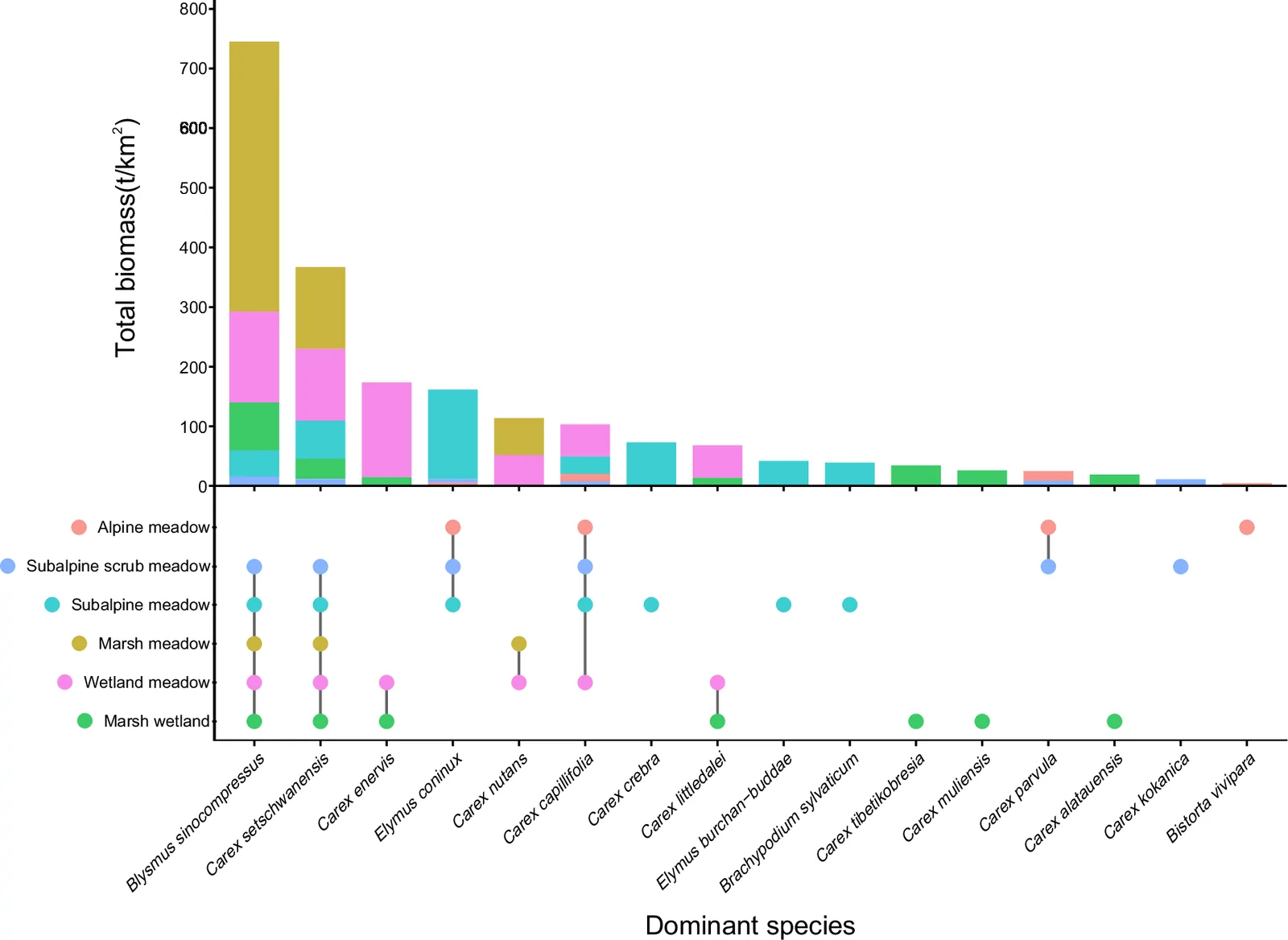
Overlap of the dominant plant species among the different grassland types.

We conducted a cross-analysis of the frequency of occurrence of dominant species in various grassland types and constructed an upset diagram to analyse, which species are widely distributed as “core dominant species” and which are “specialized species” or “indicator species” specific to a particular grassland type, thus revealing the structural similarities and differences and biodiversity patterns of different grassland communities. The dominant species compositions of different grassland types significantly overlaped, reflecting the ecological connectivity and species co-occurrence phenomena along the environmental gradient in alpine grasslands (Fig. 3).

Among the 6 grassland types, subalpine meadow and marsh wetland had 7 dominant species, followed by subalpine scrub meadow and wetland meadow with 6 dominant species each. Other grassland types (alpine meadow and marsh meadow) had 3 to 4 dominant species each. This shows that the number of dominant species decreases under colder and wetter conditions, while the number of dominant species increases under moderately humid conditions, and the area covered by these species is also larger. The number of dominant species in all types of plots showed a unimodal distribution with varying hydrothermal conditions.

Among the dominant species (Table 4), 2 plant species (*Blysmus sinocompressus*, and *Carex setschwanensis*) were dominant in 5 grassland types (excluding alpine meadow) and had the greatest biomass, indicating that they are widely distributed and have high biomass in Zoige. *Carex capillifolia* and *Carex coninux* were dominant in the 4 and 3 grassland types, respectively, indicating that these species maintain high competitiveness and high biomass contributions under different water and nutrient conditions, and can be considered as widely distributed species. 4 plant species (*Elymus nutans*, *Carex enervis*, *Carex littledalei*, and *Carex parvula*) were dominant in 2 grassland types, while 8 species were dominant in one grassland type. These findings indicate significant differences in the distribution patterns of dominant species among different grassland types, with some habitat specialization leading to variations in water availability, soil conditions, and altitudinal gradients. For example, wetland-related grasslands are dominated by Cyperaceae and Juncaceae species, whereas alpine meadow contain a greater proportion of hardy meadow plants. The six grassland types exhibit a pattern of “shared core dominant species combined with habitat-specific dominant species”, highlighting both compositional continuity and ecological differentiation across the Zoige grassland landscape. These results demonstrate that the distribution of dominant species is shaped by environmental filtering along ecological gradients, resulting in both substantial species overlap and distinct grassland-type-specific assemblages.

**Table 4:** Details of the overlap in dominant plant species among different grassland types.

Shared species	Number of grassland types	Grassland types where present
*Blysmus sinocompressus*	5	Marsh meadow; Wetland meadow; Subalpine meadow; Subalpine scrub meadow; Marsh wetland
*Carex setschwanensis*	5	Marsh meadow; Wetland meadow; Subalpine meadow; Subalpine scrub meadow; Marsh wetland
*Carex capillifolia*	4	Wetland meadow; Subalpine meadow; Subalpine scrub meadow; Alpine meadow
*Carex coninux*	3	Subalpine meadow; Subalpine scrub meadow; Alpine meadow
*Elymus nutans*	2	Marsh meadow; Wetland meadow
*Carex enervis*	2	Wetland meadow; Marsh wetland
*Carex littledalei*	2	Wetland meadow; Marsh wetland
*Carex parvula*	2	Subalpine scrub meadow; Alpine meadow
*Carex crebra*	1	Subalpine meadow
*Carex alatauensis*	1	Marsh wetland
*Carex tibetikobresia*	1	Marsh wetland
*Carex muliensis*	1	Marsh wetland
*Bistorta vivipara*	1	Alpine meadow
*Elymus burchan-buddae*	1	Subalpine meadow
*Brachypodium sylvaticum*	1	Subalpine meadow
*Carex kokanica*	1	Subalpine scrub meadow

### Grassland biomass analysis

**Fig. 4:**
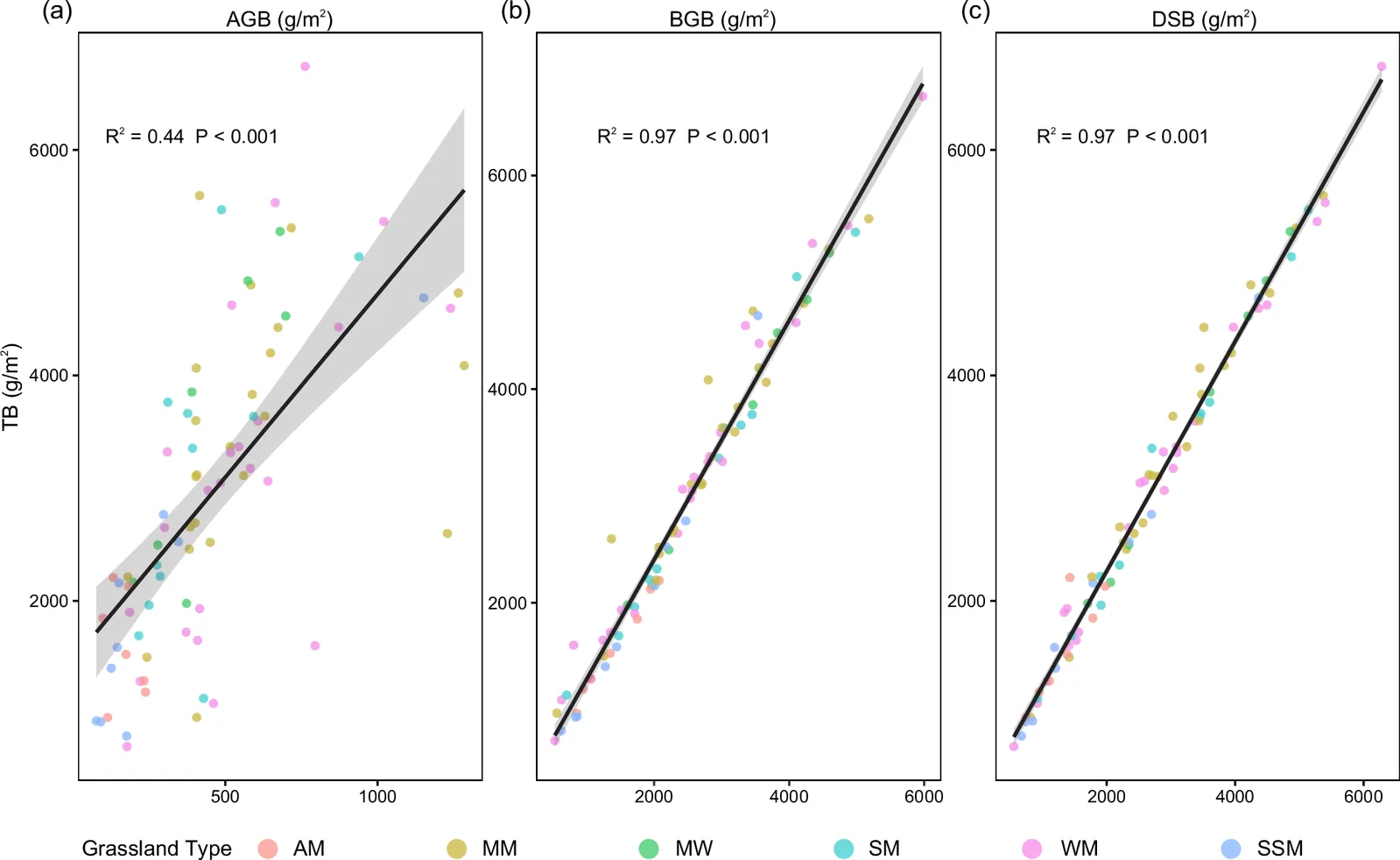
Relationships between the total biomass (TB) and the aboveground biomass (AGB), and between the belowground biomass (BGB) and the dominant species biomass (DSB) for the six grassland types in Zoige grassland. Each dot represents a plot, and the lines represent the least squares regression line. The grey areas represent the 95% confidence intervals, and different colours represent different grassland types. (AM = Alpine meadow, MM = Marsh meadow, MW = Marsh wetland, SM = Subalpine meadow, SSM = Subalpine scrub meadow, WM = Wetland meadow).

Table 5 presents descriptive statistics on the biomass parameters for each grassland type and assesses the contributions and predictive power of aboveground biomass, belowground biomass, and dominant species biomass to total biomass. Despite significant differences in biomass among the plots, the tabular data and corresponding graphical analyses (Fig. 4b,c) revealed very strong and highly significant correlations between the belowground biomass and the dominant species biomass and total biomass (R^2^ = 0.97; P < 0.001). The results demonstrate strong statistical relationships between the belowground biomass, dominant species biomass, and total biomass at the plot scale. When complete species-level sampling is not feasible, dominant species biomass measurements may provide a useful proxy for estimating total biomass patterns within sampled plots. From an ecological perspective, the grassland biomass in Zoige is dominated by root biomass, with relatively less aboveground biomass. This biomass allocation pattern enhances the adaptation of plants to the high elevation, cold environment of this region. The ratio of aboveground to belowground biomass in different grassland types further suggests that areas at higher elevations and in colder climates allocate a greater proportion of biomass belowground. This pattern is consistent with previous findings that showed that cold, arid and high-elevation conditions promote an increase in belowground biomass, which enhances the absorption of water and nutrients^33^.

**Table 5:** Biomass allocation and regression relationships among different grassland types.

Grassland type	AGB (g m^−2^)	BGB (g m^−2^)	DSB (g m^−2^)	TB(g m^−2^)	AGB → TB		BGB → TB		DSB → TB	
					R^2^	p value	R^2^	p value	R^2^	p value
Alpine meadow	166.8 ± 55.5	1,426.9 ± 498.5	1,339.3 ± 442.8	1,593.7 ± 479.5	0.084	0.528	0.989	<0.001	0.755	0.011
Marsh meadow	572.6 ± 302.1	2,843.5 ± 1,078.6	3,259.4 ± 1235.5	3,416.1 ± 1,160.5	0.153	0.065	0.934	<0.001	0.951	<0.001
Marsh wetland	455.6 ± 197.1	3,134.7 ± 1,193.0	3,677.4 ± 1,504.1	3,590.3 ± 1,364.7	0.789	0.0075	0.994	<0.001	0.995	<0.001
Subalpine meadow	413.9 ± 206.8	2,700.1 ± 1,255.6	2,947.6 ± 1,425.6	3,114.0 ± 1,380.4	0.424	0.0299	0.984	<0.001	0.976	<0.001
Subalpine scrub meadow	284.2 ± 337.4	1,692.5 ± 944.7	1,815.2 ± 1,280.9	1,976.7 ± 1,242.7	0.834	<0.001	0.979	<0.001	0.989	<0.001
Wetland meadow	545.1 ± 263.5	2,571.3 ± 1,396.6	2,968.7 ± 1,660.1	3,116.4 ± 1,557.0	0.438	<0.001	0.98	<0.001	0.972	<0.001

### Soil analysis

**Fig. 5:**
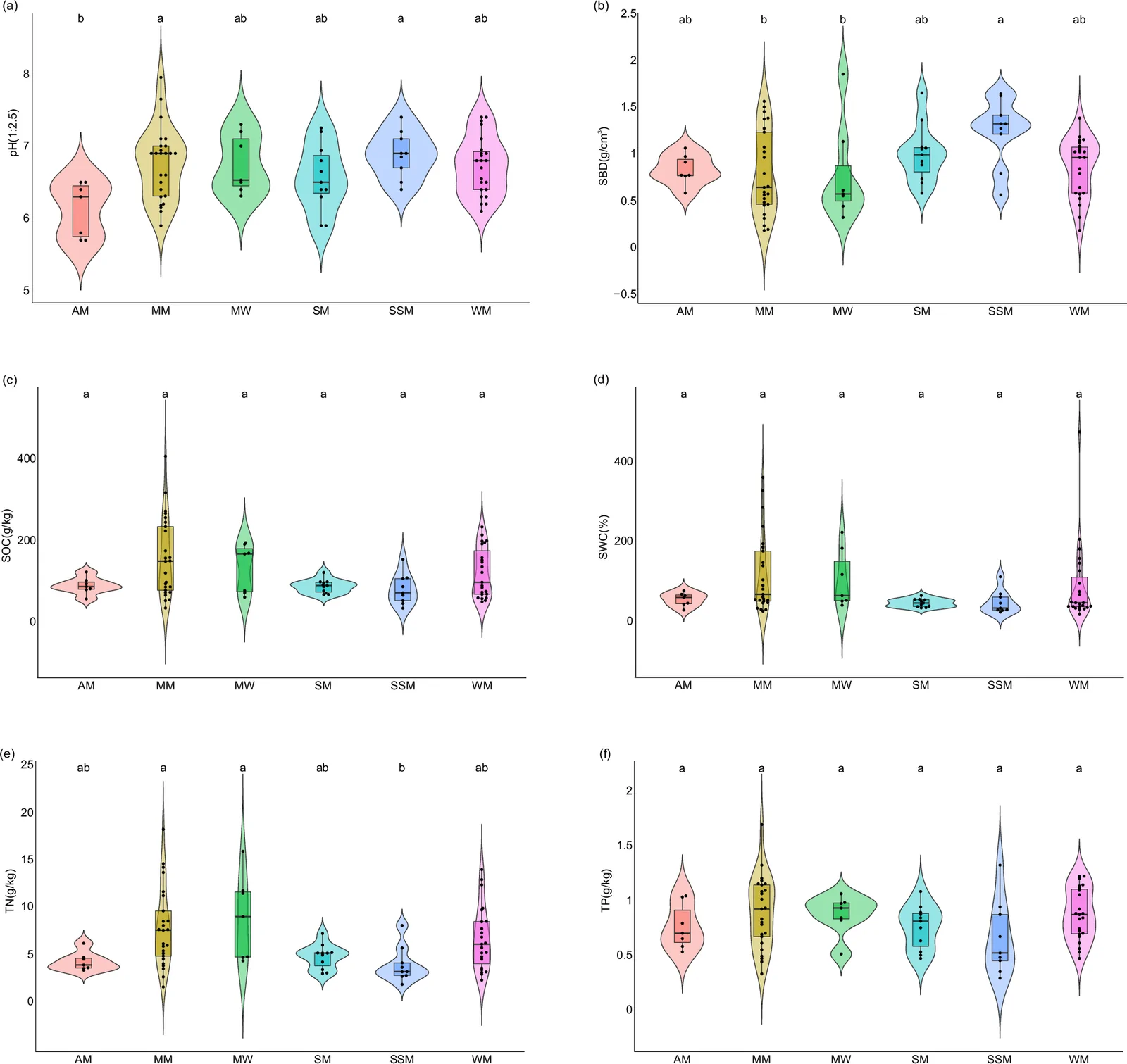
Soil pH (**a**), soil bulk density (**b**), soil organic carbon (**c**), soil water content (**d**), soil total nitrogen (**e**), and soil total phosphorus (**f**) values of all 80 plots in different grassland types. (Different lowercase letters indicate significant differences among groups, P < 0.05. Groups sharing at least one common letter are not significantly different, P ≥ 0.05).

As shown in Fig. 5, the soil physicochemical properties varied considerably among the different grassland types. With respect to soil pH (Fig. 5a), all grassland types except alpine meadow presented values within the weakly acidic to neutral range (approximately 6.0–7.5), whereas alpine meadow showed more pronounced acidification, with the soil pH decreasing to 5.7. These patterns indicate only slight fluctuations in soil acidity across most habitats, likely driven by differences in elevation and temperature. The soil bulk density (Fig. 5b) exhibited one of the clearest habitat differences, reaching its highest values in subalpine scrub meadow (1.3–1.6 g/cm^3^), reflecting a dense soil structure and low porosity. In contrast, the bulk of the densities of the marsh meadow and marsh wetland were exceptionally low (0.18–0.35 g/cm^3^), which is consistent with waterlogged soils rich in undecomposed organic residues. SOC and TN (Fig. 5c,e) also differed markedly among the grassland types. The greatest amount of SOC accumulated in the marsh meadow and marsh wetland areas, often exceeding 200 g/kg and reaching 405 g/kg, which was accompanied by elevated TN levels (>10 g/kg). In comparison, the alpine and subalpine meadow remained nutrient poor, with SOC concentrations generally less than 120 g/kg and TN concentrations between 3 and 6 g/kg. These patterns suggest that wetter habitats promote strong organic matter accumulation and nitrogen retention. This trend was reinforced by the SWC (Fig. 5d), which varied by more than an order of magnitude across grasslands—marsh systems exhibited extreme water contents (180–470%), whereas subalpine scrub meadow often remained below 35%, indicating substantial differences in water-holding capacity among habitats. TP (Fig. 5f) showed minimal variation across grassland types (0.35–1.32 g/kg), suggesting that total phosphorus is weakly linked to the vegetation type or hydrological conditions. In contrast, the N/P ratio was closely correlated with TN rather than TP, reaching high values in nutrient-rich marsh sites (>15) and remaining low (<7) in alpine and subalpine meadow, which was consistent with the similar distribution patterns observed between TN and N/P.

### Dominant species biomass analysis

**Fig. 6:**
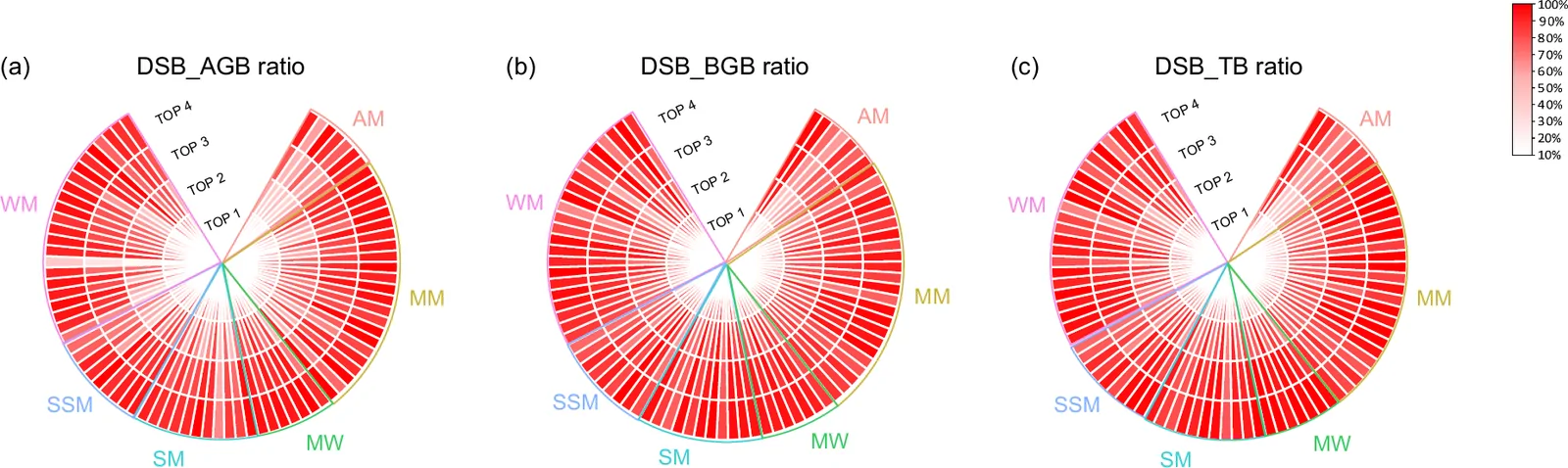
Heatmap of the cumulative biomass contributions of the dominant species (from the inner to outer rings), representing the change in the proportion of cumulative biomass to total biomass as the top-ranked species are added. (**a**) Aboveground biomass proportion, (**b**) belowground biomass proportion, (**c**) total biomass proportion. (AM = Alpine meadow, MM = Marsh meadow, MW = Marsh wetland, SM = Subalpine meadow, SSM = Subalpine scrub meadow, WM = Wetland meadow).

The cumulative contributions of the dominant species (top 1–4) to the aboveground biomass, belowground biomass, and total biomass varied across the different grassland types, but the results consistently indicated that a few species held a relative advantage (Fig. 6). Among the six grassland types, except for alpine meadow, the top-ranked biomass species (AGB, BGB, and TB) accounted for nearly half of the total biomass. The top two species contributed more than 80% of the total BGB and TB, while the top three species contributed more than 80% of the AGB. In contrast, the biomass of the dominant species in alpine meadow was less concentrated across species. Therefore, this highly uneven biomass distribution pattern suggests that the biomass of most grassland types is primarily contributed by a few dominant species, suggesting that the distribution of biomass within many grassland types is concentrated among a relatively small number of dominant species.These dominant species contribute substantially to the community biomass composition across the sampled grasslands. Conversely, the distribution of biomass among species in alpine meadow was more balanced, suggesting that these species may have stronger ecological recovery capabilities and stability in high-elevation environments. Overall, dominant species play crucial roles in regulating biomass allocation and maintaining ecosystem function.

### Dominant plant species list

We provide a collection of photos of the dominant plant species(Table 6). This collection includes photos of the habitats of the dominant species and their names. The photos are organized into folders by number and are in JPG format.

**Table 6:** List of dominant species.

Category	Species name	Family	Genus
1	*Blysmus sinocompressus*	Cyperaceae	*Blysmus*
2	*Carex setschwanensis*	Cyperaceae	*Carex*
3	*Carex enervis*	Cyperaceae	*Carex*
4	*Carex coninux*	Cyperaceae	*Carex*
5	*Carex capillifolia*	Cyperaceae	*Carex*
6	*Carex crebra*	Cyperaceae	*Carex*
7	*Elymus nutans*	Poaceae	*Elymus*
8	*Carex littledalei*	Cyperaceae	*Carex*
9	*Carex alatauensis*	Cyperaceae	*Carex*
10	*Carex tibetikobresia*	Cyperaceae	*Carex*
11	*Carex muliensis*	Cyperaceae	*Carex*
12	*Bistorta vivipara*	Polygonaceae	*Bistorta*
13	*Elymus burchan-buddae*	Poaceae	*Elymus*
14	*Brachypodium sylvaticum*	Poaceae	*Brachypodium*
15	*Carex kokanica*	Cyperaceae	*Carex*
16	*Carex parvula*	Cyperaceae	*Carex*

## Technical Validation

### Taxonomic validation

Numerous different people were involved in the 3-year data collection period, which increased the risk of observational errors in the dataset. Specifically, species may be misidentified in community or trait data (especially in the Poaceae and Cyperaceae families), which may result in some dominant species being misidentified and thus affecting grassland assessments. Before publication, we rescreened all the species photographed in the plots to detect and correct errors in species identification in the plot data.

### Data verification

We conducted a thorough check and verification of all the data, and the specific process is as follows. First, we checked and corrected missing or incorrect sample identification in the measurement by comparing the field records and the records on the envelopes containing the plants. Second, we checked and corrected the data for unrealistically high or low values and corrected the misspellings by comparing the laboratory and field records. Some data with obvious measurement errors (such as numerical errors or mismatches during recording) were eliminated. The data outliers were then rechecked by plotting the data. In addition, some leaves were very small and the dry mass may have been close to the lower limit of the balance weight sensitivity. In this case, the data were deleted.

## Usage Notes

This dataset provides a basic data resource for accurately estimating grassland biomass in the Zoige, and provides a series of supporting datasets that can provide data support for larger-scale scientific research. In addition, we used standardized and verified methods for collecting plants and soil and conducted multiple rounds of verification of the dominant species to ensure the accuracy of the data. In addition, biomass data may provide important data support for the assessment of grassland carbon sinks.

However, several limitations should be considered when using these dataset. First, measurement uncertainties are unavoidable, especially regarding the spatial and temporal representativeness of the sampling design. Spatially, some areas are difficult to access, which may result in a less than homogeneous spatial distribution of the sampled plots.In addition, although the sampling plots were preferentially established in relatively undisturbed habitats, varying degrees of grazing disturbance may still have occurred because of the widespread pastoral activities in Zoige. Therefore, potential anthropogenic influences should be considered when interpreting the vegetation productivity and community characteristics. In terms of timing, although the soil and vegetation sampling was conducted primarily during the peak of biomass period to minimize the impact of interannual fluctuations, seasonal variations in biomass may still have introduced temporal uncertainty into the dataset.

## Supplementary information

Supplementary Information

## Data Availability

This study did not involve the development or use of any custom code or scripts for data generation or processing. All calculations, analyses, and visualizations described herein were performed using commercial software.
